# The clinical practice of palliative sedation in patients dying from COVID-19: a retrospective chart review

**DOI:** 10.1186/s12904-023-01156-x

**Published:** 2023-04-04

**Authors:** Maaike Rijpstra, Evelien Kuip, Jeroen Hasselaar, Kris Vissers

**Affiliations:** 1 Department of Pain, Anaesthesiology and Palliative Medicine, Radboud University Medical Centre, Radboud Institute for Health Sciences, Nijmegen, The Netherlands; 2 Department of Primary and Community Care, Radboud University Medical Centre, Radboud Institute for Health Sciences, Nijmegen, the Netherlands

**Keywords:** COVID-19, Palliative sedation, Terminally ill, End-of-life care, Hospital inpatients, Retrospective chart review

## Abstract

**Background:**

Clinical experts experienced challenges in the practice of palliative sedation (PS) during the COVID-19 pandemic. Rapid deterioration in patients’ situation was observed while the indications for starting PS seemed to differ compared to other terminal patients. It is unclear to which extent clinical trajectories of PS differ for these COVID patients compared to regular clinical practice of PS.

**Objectives:**

To describe the clinical practice of PS in patients with COVID versus non-COVID patients.

**Methods:**

A retrospective analysis of data from a Dutch tertiary medical centre was performed. Charts of adult patients who died with PS during hospitalisation between March ’20 and January ‘21 were included.

**Results:**

During the study period, 73 patients received PS and of those 25 (34%) had a COVID infection. Refractory dyspnoea was reported as primary indication for starting PS in 84% of patients with COVID compared to 33% in the other group (p < 0.001). Median duration of PS was significantly shorter in the COVID group (5.8 vs. 17.1 h, p < 0.01). No differences were found for starting dosages, but median hourly dose of midazolam was higher in the COVID group (4.2 mg/hr vs. 2.4 mg/hr, p < 0.001). Time interval between start PS and first medication adjustments seemed to be shorter in COVID patients (1.5 vs. 2.9 h, p = 0.08).

**Conclusion:**

PS in COVID patients is characterized by rapid clinical deterioration in all phases of the trajectory. What is manifested by earlier dose adjustments and higher hourly doses of midazolam. Timely evaluation of efficacy is recommended in those patients.

## Background

During the COVID pandemic, there were different waves of reported patients with this infection. Although most patients had mild respiratory symptoms, in approximately 15% of the cases hospitalization was needed while in 5% the symptoms developed to a critical disease [1]. Increase in severity of illness was observed when dyspnoea was a present symptom [1].

Symptom burden at the end of life in patients with COVID is mainly due to breathlessness and agitation [2]. These symptoms are often treated with low doses of opioids and benzodiazepines in COVID and non-COVID patients alike. Responsiveness to these treatments was reported as equally effective in these 2 groups in the study of Alderman [3].

Although, the management of symptoms at the end of life is in general the same as for other terminally ill patients, data are scarce for COVID patients with a deteriorating situation nearing death. In some patients at the end of life, conventional modes of treatments fail to relieve their symptom burden within an acceptable timeframe. Palliative sedation (PS) may be considered as a last resort option to provide comfort for those dying patients [4].

Regarding palliative sedation for dying COVID patients, several challenges were noted by clinical experts in the first wave of COVID infections [5]. Health care teams were challenged by rapid deterioration of symptom burden and higher frailty scores occurred in cases with progressive COVID infection without therapeutic responses on the chosen treatment strategies. Therefore, the hypothesis is that clinical trajectories of PS in hospitalized patients dying from COVID might vary in their presentation, timeframes and used dosages of sedatives from PS trajectories of non-COVID infected patients.

Differences in baseline characteristics (age, presence of comorbidities, higher frailty scores, prescription of opioids and symptom burden) probably lead to other start- and cumulative dosages of medication compared to non-COVID patients. Importantly, the mean total duration of PS might vary between COVID and non-COVID patients. Besides, time for decision making is likely to be shorter due to the short time duration between hospital admission and initiation of PS in critical COVID patients. Hence, the involvement of palliative care specialists in the care process might be decreased.

We conducted a retrospective monocentre analysis of patient medical records, to describe the clinical course of PS in patients dying from COVID. Secondly, we compared this clinical course of PS with the course of patients dying from other diagnoses during the same time period. The results of this research can facilitate the development of better clinical guidelines that might support health care professionals in a more tailored PS trajectory in patients dying from acute infectious diseases like COVID-19.

## Methods

### Design, setting and sample

Data of deceased adult (≥ 18 years) patients were collected retrospectively between 1st of March ’20 and 31st of December ’20 based on whether they received PS at one of the hospital wards in a Dutch tertiary medical centre (Radboudumc, Nijmegen).

Data of patients admitted at one of the Intensive Care Units (ICU) at time of death were excluded from this analysis since these patients used sedative medication for other medical reasons related to their intensive care treatment and without refractory symptoms. Patients with a registered positive test for COVID-19 or a clinical diagnosis based on other diagnostics were analysed in the COVID group while patients without a COVID diagnosis served as reference group.

### Data collection

Data was collected from the electronic patient database (EPIC). Independent data managers extracted pseudonymized data for all deceased patients hospitalized at the Radboudumc during the study period. Patient records were filtered for administration of sedative medication (e.g., midazolam or levomepromazine) during the last admission. Patients who did not receive sedative medication in the last 24 h of life were excluded from the database. The remaining records were checked for the aim of sedative medication. Patient records were included for analysis when in the records explicitly was stated that the aim of sedative medication was PS. Records without explicitly stated aim of PS were discussed with two independent clinical palliative experts and cases were included when those experts unanimously decided that administration of sedatives was due to PS.

The charts of eligible participants were checked for baseline characteristics: gender, age, primary reason admission, diagnoses and co morbidities. Also, several variables were collected for three different time periods regarding the PS trajectories:

1. Before the start of PS: involvement of the palliative care team (PCT); amount of time between admission and starting PS; the main indications; administration of opioids during hospitalization.
2. At the start of PS: sort, dosage and administration route of sedative medication; decision regarding administration of opioids.
3. During PS: changes in medication sort, dosage, and timing for those adjustments; changes in opioids; total duration of PS; cumulative doses of medication during PS.

### Analysis

For statistical analysis, SPSS version 25 (SPSS, 2017, Inc. Chicago, IL) and R software version 3.6.0 (The R Foundation for Statistical Computing, Vienna, Austria) were used. Missing data were explored, reasons for missing data were reported and when missing at random data was imputed with multiple regression imputation [6].

For the descriptive analysis, numbers and proportions for categorical variables were used and mean and standard deviations for continuous variables, and medians with interquartile ranges were calculated for ordinal data or data with a skewed distribution. Differences between groups were tested using a Chi-square test for categorical variables (Fischer’s exact test in case of cell frequencies < 5), a Kruskal-Wallis test for ordinal variables, and ANOVA for continuous variables.

After the analysis of patients receiving PS during the COVID pandemic, a sensitivity analysis was performed with a control group of patients. For this control group data were included with the same criteria, but for patients hospitalized in the time period prior to the pandemic (1st of August ’19- 29th of February ’20). In this analysis two groups were used based on the hospitalization period. The control group was compared with the total sample (COVID and non-COVID cases) hospitalized during the COVID-19 pandemic. This analysis was performed to ensure differences in clinical- and PS courses were not caused by differences in the hospitalization period during COVID.

## Results

### Population

During the first waves of the COVID pandemic in 2020, a total group of 73 patients received palliative sedation because of refractory symptoms before dying. Of those, 25 patients (34%) had a confirmed COVID infection while 48 (66%) died of other diagnoses. Patients who died of COVID tend to be older (mean 74y vs. 69y, p 0.07). Besides, 88% of the COVID patients had at least one other present diagnose at admission of which a cardiovascular disease was less likely (4% vs. 31%, p < 0.01). All baseline characteristics are presented in Table 1.

### Before the start of PS

No significant difference between the groups was found for the median duration of time between admission to the hospital and the start of PS (5.8 days vs. 3.6 days respectively, p 0.1). In 84% of patients with COVID the primary indication for starting PS was refractory dyspnoea compared to 33% in the non-COVID group (p < 0.01).

In approximately 90% of both groups (n = 23 vs. n = 43) administration of opioids was started before the initiation of PS. Morphine was the predominantly administered opioid with dyspnoea, pain and/or discomfort as the main indications for prescription. In the 24 h before start of PS, the cumulative dose of administered morphine was 21.2 mg [13.0–34.0 mg] in the COVID- vs. 15.0 mg [8.7–25.2 mg] in the non-COVID group (p = 0.2).

**Table 1:** Baseline characteristics of patients with- and without COVID-19

	COVID	Non-COVID	
Characteristics	N = 25 (%)	N = 48 (%)	p-value
Gender, male	14 (56)	31 (65)	0.5
Age, yrs., mean (SD)	74 (8.2)	69 (14.9)	0.07
Diagnoses present, at admission^a^			
Malignancy	7 (28)	21 (44)	0.2
COPD	6 (24)	8 (17)	0.5
Cardiovascular disease	1 (4)	15 (31)	**< 0.01**
Infectious disease	25 (100)	10 (21)	**< 0.001**
Other	11 (44)	17 (35)	0.5
Number of present diagnoses			
1	3 (12)	30 (63)	**< 0.001**
2 or more	22 (88)	18 (38)	**< 0.001**
Comorbidity present, at admission^b^			
Hypertension/CHF	13 (52)	24 (50)	0.9
Dementia	3 (12)	1 (2)	0.08
Respiratory. Disease	6 (24)	4 (8)	0.06
Diabetes Mellitus	8 (32)	12 (25)	0.5
Renal Failure	2 (8)	5 (10)	0.7
Other	4 (16)	7 (15)	0.9
Number of present comorbidities			
0	3 (12)	17 (35)	**< 0.05**
1	12 (48)	15 (31)	0.2
2 or more	10 (40)	16 (33)	0.6

COPD = Chronic Pulmonary Disease, CHF = Congestive Heart Failure

^a^ Diagnoses: More than one diagnosis could be present at admission. Other diagnoses such as neurological disorders, Rheuma

^b^ Comorbidities: Patients could have been diagnosed with multiple comorbidities. Other comorbidities such as pulmonary embolism, liver failure

The palliative care team (PCT) was involved in approximately 65% of both groups during the last days of life (n = 17 vs. n = 30, p 0.4). There were significant differences in the timing of the first bedside consult by the PCT. In 12 COVID patients (48%) a bedside consult by the PCT was performed before the start of PS while 4 other patients of this group (20%) were visited after the start of PS. Non-COVID patients were visited by the PCT respectively in 58% before start- and 4% after start of PS. See Table 2. for the palliative sedation variables.

**Table 2:** Comparison of variables related to PS of patients with- and without COVID-19

	COVID	Non-COVID	
Variables (n (%) or median [IQR])	N = 25 (%)	N = 48 (%)	p-value
PCT Consults, yes	17 (68)	30 (62)	0.4
First consult before start PS	12 (48)	28 (58)	**< 0.05**
First consult after start PS	5 (20)	2 (4)	**< 0.05**
Consults both before and after start	1 (4)	5 (10)	0.3
Time (in days) between admission and start PS	5.8 [3.6–10.8]	3.6 [1.3–9.3]	0.1
Primary reason to start PS			
Dyspnoea	21 (84)	16 (33)	**< 0.001**
Terminal Restlessness/Agitation	2 (8)	17 (35)	**< 0.05**
Other (Pain, Delirium/Anxiety etc.)	2 (8)	15 (31)	**< 0.05**
Increase of medication after start, yes	19 (76)	35 (73)	0.8
Time (in hrs) between start PS and first increase	1.5 [0.9–2.7]	2.9 [1.1–7.3]	0.08
Time duration PS in total (in hours)	5.8 [2.2–19.5]	17.1 [7.7–33.4]	**< 0.01**
(Mean (SD))	13.6 (21.2)	25.7 (32.8)	0.06

PCT = palliative care team, PS = palliative sedation, IQR = interquartile range, SD = standard deviation

### Start of PS

At the start of PS, most patients in both groups received a bolus- and continuous infusion of midazolam was started subsequently (52% vs. 69%, p 0.2). Approximately a third of the COVID group started PS with a single bolus of midazolam only (36% vs. 25%, p 0.3).

No differences were found in start doses of bolus- and continuous infusion of midazolam. The median cumulative midazolam dose administered in the first hour of PS was 5.0 mg for both the COVID- and the non-COVID group.

Administration of morphine was continued in 21 (84%) COVID- and 39 (81%) non-COVID patients (p 0.8).

**Table 3:** Medication during palliative sedation for patients with- and without COVID-19.

	COVID		Non-COVID			
Variables (n (%) or median [IQR])	N = 25 (%)		N = 48 (%)		p-value	
Midazolam form, at start PS						
Single Bolus		9 (36)		12 (25)		0.3
Continuous infusion only		3 (12)		3 (6)		0.4
Both bolus and continuous infusion		13 (52)		33 (69)		0.2
Midazolam initial doses						
Start dose bolus (mg)		5.0 [2.5–10.0]		5.0 [2.5-5.0]		0.17
Start rate continuous infusion (mg/hr.)		1.5 [1.5–1.5]		1.0 [1.0-1.5]		0.06
Total initial dose (mg) in first hour of PS		5.0 [2.5–11.0]		5.0 [3.0-6.5]		0.4
Midazolam doses during the total PS period						
Cumulative dose (in mg) during PS (Mean (SD))		19.0 [12.2–49.8] 51.9 (91.7)		30.4 [13.9–64.1] 79.2 (163.2)		0.4 0.4
Recalculated hourly dose (in mg/hr) (Mean (SD))		4.2 [3.7–5.8] 5.2 (3.7)		2.4 [1.4–3.7] 2.7 (1.7)		**< 0.001** **< 0.01**
Morphine						
Morphine prescribed at start PS, yes		23 (92)		43 (90)		1.0
Cumulative dose (in mg) day before start PS		21.2 [13.0–34.0]		15.0 [8.7–25.2]		0.2
Morphine during PS, yes		22 (88)		44 (91)		0.8
Recalculated hourly dose (mg/hr) during PS		2.1 [1.6–3.6]		2.0 [1.1–3.1]		0.5

PS = palliative sedation, IQR = interquartile range, SD = standard deviation

### During PS

In both groups a comparable percentage of patients (76% vs. 73%) needed an increase of sedative medication once PS was started. The median time until the first increase was 1.5 h in the COVID group and 2.9 h in the non-COVID group (p 0.08). In most patients with the need for rescue medication, a bolus was administered and the rate of continuous infusion of midazolam was increased (56% vs. 52%).

The total cumulative dosages of midazolam were recalculated to hourly dosages of midazolam during the total PS period. This was significantly higher in the COVID group (median 4.2 mg/hour vs. 2.4 mg/hour, p < 0.001).

The median hourly dosage of morphine during PS was equal for the patients with- or without COVID (2.1 mg/hr [1.6–3.6] vs. 2.0 mg/hr [1.1–3.1], p 0.5). All medication variables are shown in Table 3.

### End of PS

**Fig. 1:**
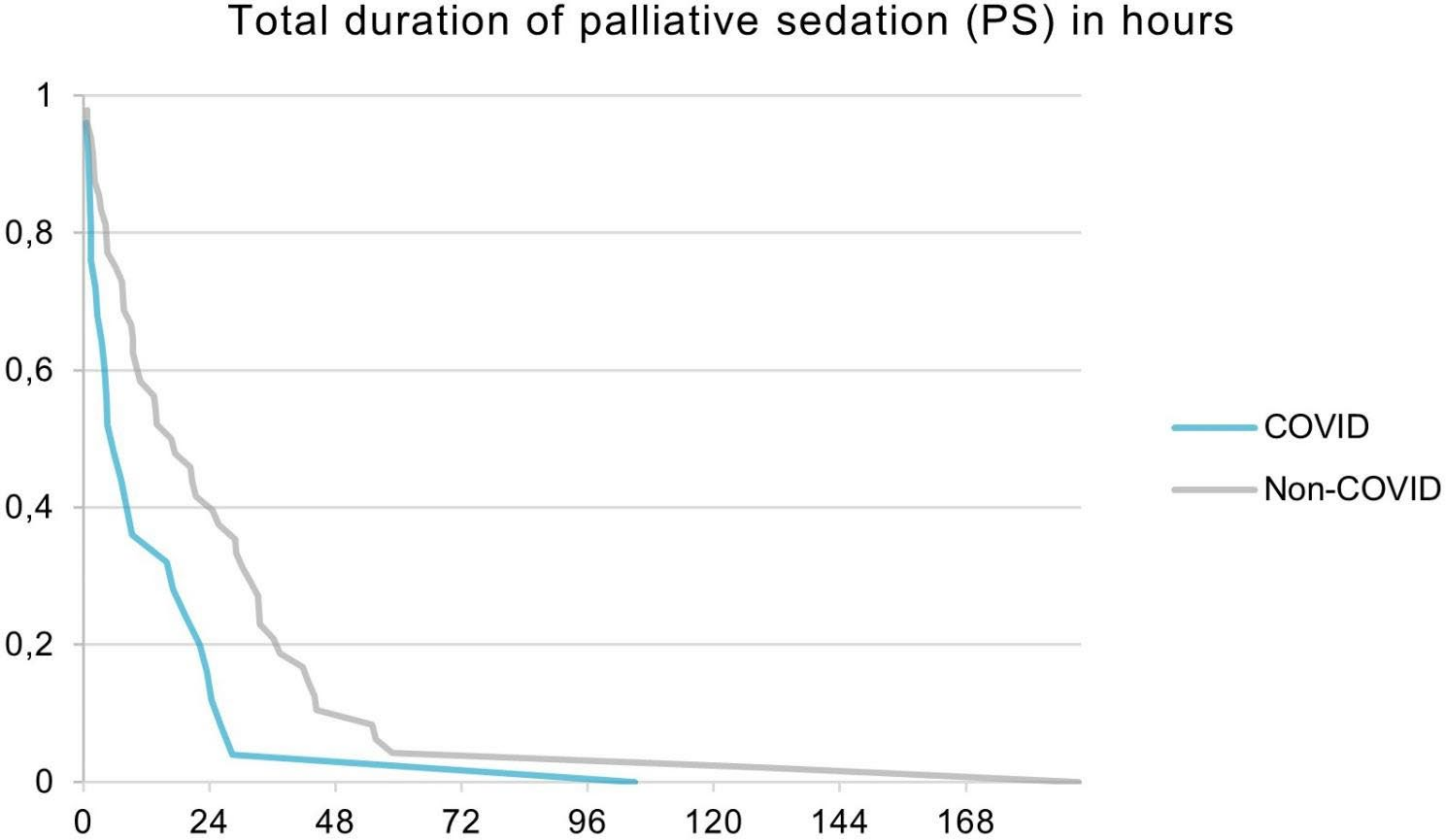
Survival Curve during PS for the COVID (n = 25) and non-COVID patients (n = 48)

The total duration of PS was significant shorter for the COVID patients with a median of 5.8 h [2.2–19.5] compared to 17.1 h [7.7–33.4] for the non-COVID patients (p < 0.01). (See Fig. 1.)

### Hospitalization period

For the sensitivity analysis, the total group of 73 patients hospitalized during the pandemic was compared with a group of 58 patients that received PS during hospitalization before the pandemic. No significant differences were found for the baseline characteristics and/or palliative sedation variables e.g., regarding dosages and duration. However, there was a significant difference in the number of patients which were visited by the PCT both prior to start- and during PS. In 8% (n = 6) of the group hospitalized during the pandemic there were consults of the PCT before- and after start of PS while in the pre-pandemic group this was 22% (n = 13, p < 0.05).

## Discussion

This study confirms our original hypothesis that palliative sedation trajectories of COVID patients were influenced by their rapid deteriorating situation. We conclude there were agreements in the trajectories of PS (e.g., starting form) while other variables differed between COVID and non-COVID patients. This is shown by significant higher doses of medication, faster need to increase medication and shorter duration of sedation. This research adds information about the trajectories of dying COVID patients at regular wards which is scarce since most data are about patients dying at high- or intensive care wards.

One of our findings is the fast deterioration of the patients’ situation followed by initiation of PS within a week (median) after admission to the hospital. This result compares well with the observed time between admission- and deterioration of the patients with referral to palliative care [2, 7]. Ramos et al. conclude in their study that the late start of palliative sedation in their COVID population might be due to the lack of specialized palliative care involvement in the process [8].

In our study we also investigated the involvement of palliative care teams (PCT). Involvement of a PCT is considered important to improve the quality of care [9]. Especially when palliative care, of e.g. palliative sedation, must be provided by professionals with minor experience in this care [9, 10]. The involvement of PCT in our study was well balanced between the COVID- and non-COVID patients. However, timing of the first- and frequency of PCT consultation varied between the COVID and non-COVID groups. The COVID group had less consultations before start of PS and more often a single consultation. These results are in line with a previous study mentioning less frequent and less intense involvement of PCT consultations in COVID patients [7]. Differences in PCT consultation were also seen in the sensitivity analysis comparing PCT involvement before and during the pandemic. Part of these differences might be explained by fast organisational changes in daily clinical care with high workload and probably decreased attention for palliative care. However, most differences are found between COVID and non-COVID patients which is presumably explained by the rapid deterioration of the COVID patients. The prognostic uncertainty about the dying phase in COVID patients together with a rapid worsening in condition might hamper timely involvement of the PCT before start of PS. Additionally, the shorter dying phase offers less time- and probably therefore less frequent PCT consultations.

We explain the shorter PS duration for the COVID patients in our study as an expected result caused by their disease and symptom burden. The shorter dying phase for the COVID patients was shown in previous research as well [7]. Besides, dyspnoea was reported as a predictive factor for mortality in COVID patients [11]. At last, shorter survival time was also observed in other terminal patients with refractory dyspnoea caused by infectious disease [12].

Apparently, the COVID patients of our sample needed earlier increase of medication and received higher doses of midazolam in a shorter timeframe. This finding is similar to a report of palliative care specialists in a Dutch hospital in which higher doses of sedative medication in dying COVID patients were needed to reach comfort at the end of life [13]. Opposite, the lower doses of midazolam in our study for the non-COVID group are comparable with the reported doses for the other terminally ill hospital inpatients in the study of Abdul et al [14]. Therefore, we presume that patients with COVID infection and/or refractory dyspnoea requires a higher individual dosage of midazolam. This assumption is supported by the results of the sensitivity analysis that the hospitalization period as such is not an influencing factor for differences in PS trajectories. Despite a strained health care system during the pandemic and the overwhelming workload for health care professionals, the care for COVID patients at the end-of-life still followed the national guidelines. However, based on the aforementioned results, we would recommend special attention for timely and adequate clinical monitoring of the efficacy of PS in patients with COVID or similar disease progression at the end of their life.

Several limitations of this study have to be considered. First, the retrospective method might have introduced selection bias in the in- and exclusion of patients receiving palliative sedation. In the patients without an explicitly reported aim of starting sedative medication we might have misjudged the real purpose by constructing this afterwards. Besides, we did not have the opportunity to sample data in a standardized and structured manner for all variables. Another limitation is the exclusion of ICU patients leaving a small sample of remaining COVID patients dying at the wards.

Despite, these findings will add information to support healthcare providers’ considerations about trajectories of PS in COVID, but also in other patients with rapid deteriorating situations and need for tight monitoring of the efficacy.

## Conclusion

PS in COVID is characterised by earlier dose adjustments and higher doses of midazolam per hour and a significant shorter PS duration. Additionally, all physicians involved in the care for deteriorating patients with COVID or any other life limiting disease should be aware of basic principles of appropriate PS including the adjustment of dosages and clinical monitoring since the rapid evolution of these patients reduced the timely involvement of a dedicated supportive and palliative care team before start of PS.

More research is needed about recommendations for a tailored approach of PS in patient groups with specific rapid deteriorating clinical situations such as in COVID infection.

## Data Availability

The datasets generated during and analysed during the current study are not publicly available as they were not part of the consent but are available from the corresponding author on reasonable request. Results of a preliminary analysis of the data from this study were presented at the World Congress of the EAPC 2021.[15].

## Abbreviations

PS: Palliative sedation
COVID-19: Coronavirus Disease 2019
ICU: Intensive Care Unit
PCT: Palliative Care Team

## References

[1] Talukder A, Razu SR, Alif SM, Rahman MA, Islam SMS (2022). Association between symptoms and severity of Disease in Hospitalised Novel Coronavirus (COVID-19) patients: a systematic review and Meta-analysis. J Multidiscip Healthc.

[2] Lovell N, Maddocks M, Etkind SN (2020). Characteristics, Symptom Management, and outcomes of 101 patients with COVID-19 referred for Hospital Palliative Care. J Pain Symptom Manage.

[3] Alderman B, Webber K, Davies A (2020). An audit of end-of-life symptom control in patients with corona virus disease 2019 (COVID-19) dying in a hospital in the United Kingdom. Palliat Med.

[4] Cherny NI, Radbruch L, Board of the European Association for Palliative C (2009). European Association for Palliative Care (EAPC) recommended framework for the use of sedation in palliative care. Palliat Med.

[5] Hasselaar J, Vissers K, Mercadante S et al. *Palliative sedation in the context of COVID-19: Expert opinions from the Palliative Sedation project*. EAPC. 2020: https://eapcnet.wordpress.com/2020/04/20/palliative-sedation-in-the-context-of-covid-19-expert-opinions-from-the-palliative-sedation-project/.

[6] Preston NJ, Fayers P, Walters SJ (2013). Recommendations for managing missing data, attrition and response shift in palliative and end-of-life care research: part of the MORECare research method guidance on statistical issues. Palliat Med.

[7] Hetherington L, Johnston B, Kotronoulas G (2020). COVID-19 and Hospital Palliative Care - A service evaluation exploring the symptoms and outcomes of 186 patients and the impact of the pandemic on specialist Hospital Palliative Care. Palliat Med.

[8] Ramos-Rincon JM, Moreno-Perez O, Gomez-Martinez N et al. *Palliative Sedation in COVID-19 End-of-Life Care. Retrospective Cohort Study* Medicina (Kaunas). 2021;57(9). 10.3390/medicina57090873 PMC8470831 34577796

[9] Golob S, Zilinyi R, Godfrey S (2022). The prevalence of Palliative Care Consultation in deceased COVID-19 patients and its association with end-of-Life Care. J Palliat Med.

[10] Arantzamendi M, Belar A, Payne S (2021). Clinical aspects of Palliative Sedation in prospective studies. A systematic review. J Pain Symptom Manage.

[11] Berenguer J, Borobia AM, Ryan P (2021). Development and validation of a prediction model for 30-day mortality in hospitalised patients with COVID-19: the COVID-19 SEIMC score. Thorax.

[12] Okabayashi H, Kitamura H, Ikeda S (2021). Patients with terminal interstitial pneumonia require comparable or more palliative pharmacotherapy for refractory dyspnea than patients with terminal Lung Cancer. Palliat Med Rep.

[13] ten Cate M, Brink A, Struik L, Thijs-Visser M. *Palliative Sedation for COVID 19 patients can be improved [article in Dutch]*. Medisch Contact. 2021: https://www.medischcontact.nl/nieuws/laatste-nieuws/artikel/palliatieve-sedatie-voor-covid-19-patienten-kan-veel-beter.html

[14] Abdul-Razzak A, Lemieux L, Snyman M, Perez G, Sinnarajah A (2019). Description of continuous Palliative Sedation Practices in a large Health Region and Comparison with Clinical Practice Guidelines. J Palliat Med.

[15] Abstracts from the 17th World Congress of the EAPC 2021. *Palliative Medicine.* 2021;35(1_suppl):1-243, abstract number: R-53, 10.1177/02692163211035909DOI

